# Dissecting negative effects of two root-associated bacteria on the growth of an invasive weed

**DOI:** 10.1093/femsec/fiae116

**Published:** 2024-08-22

**Authors:** Xiangyu Liu, Hocelayne Paulino Fernandes, Adam Ossowicki, Klaas Vrieling, Suzanne T E Lommen, Thiemo Martijn Bezemer

**Affiliations:** Above-Belowground Interactions Group, Institute of Biology, Leiden University, P.O. Box 9505, 2300 RA Leiden, The Netherlands; Above-Belowground Interactions Group, Institute of Biology, Leiden University, P.O. Box 9505, 2300 RA Leiden, The Netherlands; Natural Products Laboratory, Institute of Biology, Leiden University, P.O. Box 9505, 2300 RA Leiden, The Netherlands; Above-Belowground Interactions Group, Institute of Biology, Leiden University, P.O. Box 9505, 2300 RA Leiden, The Netherlands; Departamento de Microbiología, Instituto de Hortofruticultura Subtropical y Mediterránea ‘La Mayora’, Universidad de Málaga-Consejo Superior de Investigaciones Científicas (IHSM-UMA-CSIC), Universidad de Málaga, 29010 Málaga, Spain; Above-Belowground Interactions Group, Institute of Biology, Leiden University, P.O. Box 9505, 2300 RA Leiden, The Netherlands; Above-Belowground Interactions Group, Institute of Biology, Leiden University, P.O. Box 9505, 2300 RA Leiden, The Netherlands; Above-Belowground Interactions Group, Institute of Biology, Leiden University, P.O. Box 9505, 2300 RA Leiden, The Netherlands

**Keywords:** bacterial volatiles, common ragwort, concentration-dependent effects, plant–microbe interactions, root-associated bacteria, seed germination, seedling growth, weed control

## Abstract

Plant-associated microorganisms can negatively influence plant growth, which makes them potential biocontrol agents for weeds. Two Gammaproteobacteria, *Serratia plymuthica* and *Pseudomonas brassicacearum*, isolated from roots of *Jacobaea vulgaris*, an invasive weed, negatively affect its root growth. We examined whether the effects of *S. plymuthica* and *P. brassicacearum* on *J. vulgaris* through root inoculation are concentration-dependent and investigated if these effects were mediated by metabolites in bacterial suspensions. We also tested whether the two bacteria negatively affected seed germination and seedling growth through volatile emissions. Lastly, we investigated the host specificity of these two bacteria on nine other plant species. Both bacteria significantly reduced *J. vulgaris* root growth after root inoculation, with *S. plymuthica* showing a concentration-dependent pattern *in vitro*. The cell-free supernatants of both bacteria did not affect *J. vulgaris* root growth. Both bacteria inhibited *J. vulgaris* seed germination and seedling growth via volatiles, displaying distinct volatile profiles. However, these negative effects were not specific to *J. vulgaris*. Both bacteria negatively affect *J. vulgaris* through root inoculation via the activity of bacterial cells, while also producing volatiles that hinder *J. vulgaris* germination and seedling growth. However, their negative effects extend to other plant species, limiting their potential for weed control.

## Teaser

Two bacterial strains, isolated from roots of the invasive weed *Jacobaea vulgaris*, can negatively affect its growth through root inoculation and the release of volatiles, but their negative effects also extend to other plant species, limiting their potential for weed control.

## Introduction

Soil microbes play a crucial role in influencing plants (Babalola 2010, Berendsen et al. 2012, Philippot et al. 2013, Banerjee and van der Heijden 2023). They can significantly influence plant health by performing numerous functions, such as nitrogen fixation, phosphorus solubilization, and suppression of antagonists (i.e. pests and pathogens) (Hurek et al. 2002, Sessitsch et al. 2002, Carrión et al. 2019). Soil microbes can also be harmful to plants, causing diseases or competing with the plant for nutrients (Kuzyakov and Xu 2013). The critical interface where interactions between plants and soil microbes occur is referred to as the rhizosphere. In the vicinity of roots, many bacteria with various relationships to plants reside in the rhizoplane (root surface), the rhizosphere (soil surrounding the roots), and the root endosphere (inside of root tissues) (Saeed et al. 2021). These root-associated bacteria exhibit relationships with plants, ranging from mutualistic to pathogenic (Kuzyakov and Xu 2013). A central focus of this research field lies in understanding the effects of root-associated bacteria on plants and unraveling the underlying mechanisms that govern their interactions (Jacoby et al. 2017, Pascale et al. 2020, Pantigoso et al. 2022, Enagbonma et al. 2023).

Root-associated bacteria can directly influence plant performance through the production of metabolites, and the effects are often dose- or concentration-dependent (Shantharaj et al. 2021). For example, the cell-free supernatants of *Burkholderia gladioli* C101, a rhizobacterial strain, showed a dose-dependent reduction in spot disease severity caused by *Xanthomonas perforans* on tomato plants (Shantharaj et al. 2021). At high concentrations, root-associated bacteria can cause overstimulation of plant hormone production, nutrient imbalances, or produce toxins that harm plant cells or tissues (Kudoyarova et al. 2019). This can result in reduced growth, stunted development, and compromised plant health. In previous experiments, we observed that two bacteria that were isolated from the roots of the plant *Jacobaea vulgaris, Serratia plymuthica*, and *Pseudomonas brassicacearum*, had a negative impact on root growth of *J. vulgaris in vitro* (Liu et al. 2024). It is currently unclear how these bacteria influence root growth and whether these negative effects on root growth are dependent on the concentration of bacteria in the inoculum.

Except for secreting metabolites into the surrounding environment such as the vicinity of roots, many studies have found that root-associated bacteria can affect plants at a distance through volatiles (Ryu et al. 2003, Kai et al. 2010, Blom et al. 2011a, Garbeva and Weisskopf 2020, Martín-Sánchez et al. 2020). Bacterial volatiles contain both organic and inorganic compounds, and they can either promote or inhibit plant growth and seed germination (Wenke et al. 2012, Yasmin et al. 2021, He et al. 2023). For example, the volatiles produced by *S. plymuthica* have been shown to significantly inhibit growth and induce hydrogen peroxide production in *Arabidopsis* plants (Kai et al. 2010, Wenke et al. 2012). The gases hydrogen cyanide (HCN), ammonia, and dimethyl disulfide (DMDS) that can be produced by bacteria can also have inhibitory effects on plants (Kai et al. 2010, Cordovez et al. 2018). Besides influencing plant growth, volatiles of *Pseudomonas pseudoalcaligenes* have been reported to increase the percentage of germinated seeds and the germination index compared to the control treatment under a drought condition (Yasmin et al. 2021). Similar to plant–bacteria interactions through direct contact, these interactions via volatiles may also be concentration-dependent. For example, plant responses to a bacterial volatile compound, DMDS, have been found to follow a dose-dependent pattern. Cordovez et al. (2018) showed that at concentrations of 1 µM, this volatile has a significant positive effect on shoot biomass, while at concentrations higher than 1 mM it had negative effects on plant biomass. It is currently unknown whether the aforementioned two root-associated bacteria inhibit seed germination and seedling growth of *J. vulgaris* through the emitted volatiles and if so, whether these effects are concentration-dependent.

Both root-associated bacteria that are used in this study cause shortening and thickening roots of *J. vulgaris* (Liu et al. 2024). This change in root morphology resembles the response of roots to the plant hormone ethylene, as documented in previous studies (Goodlass and Smith 1979, Feldman 1984, Zaat et al. 1989). Ethylene production in plants can be induced by various abiotic and biotic factors, such as flooding and infection by Rhizobium bacteria (Zaat et al. 1989, Sasidharan and Voesenek 2015). For example, Zaat et al. (1989) reported that the ethylene inhibitor aminoethoxyvinylglycine (AVG) can restore the normal nodulation by Rhizobium bacteria in *Vicia sativa* plants without exhibiting the thick and short root phenotype. Based on this, we predict that the shorter and thicker root phenotype of *J. vulgaris* after exposure to these two root-associated bacteria may not appear in the presence of the ethylene-inhibitor AVG.

In this study, we investigated whether the effects of the bacteria, *S. plymuthica* and *P. brassicacearum*, on *J. vulgaris* through root inoculation are concentration dependent. Next, we examine whether cell-free supernatants of these bacteria can induce inhibitory effects on roots, and whether the shorter and thicker root phenotype is absent in the presence of the ethylene-inhibitor AVG. Subsequently, we conducted both *in vitro* and soil bioassays to investigate whether the two bacteria negatively affect seed germination and growth of *J. vulgaris* via emission of volatiles, and whether these effects also follow a concentration-dependent pattern. The volatile profiles of the bacteria were detected and identified by Headspace-gas chromatography-mass spectrometry (HS-GC–MS). Lastly, to further examine their potential for biological control of *J. vulgaris*, we tested the specificity of the two bacteria and tested whether the two bacteria influence the growth of other co-occurring plant species *in vitro*.

## Materials and methods

### Plants

*Jacobaea vulgaris* Geartn. subs. *vulgaris* (syn. *Senecio jacobaea* L.; Asteracaea) is a monocarpic perennial that is native in Europe (Harper and Wood 1957). Seeds were collected from plants growing in the nature reserve Meijendel near Wassenaar in The Netherlands in a coastal dune grassland. Nine other plant species that all co-occur with *J. vulgaris* in natural grasslands in The Netherlands were used in this study to examine the specificity of the two bacteria: three grasses (*Anthoxanthum odoratum* L., *Agrostis capillaris* L., *Holcus lanatus* L.), three forbs (*Plantago lanceolata* L., *Tripleurospermum maritimum* (L.) Koch, *Achillea millefolium* L.), and three legumes (*Lotus corniculatus* L., *Trifolium dubium* Sibth., *Trifolium repens* L.). Seeds of these nine species were purchased from Cruydt-Hoeck (Nijeberkoop, The Netherlands), a supplier of seeds obtained from wild plants. Seeds were surface-sterilized by submerging in a 5% sodium hypochlorite solution for 20 min, followed by rinsing three times with sterilized Mill-Q water. Subsequently, the seeds were placed in containers (10 cm × 10 cm × 4 cm), filled with sterile glass beads submerged in sterilized Mill-Q water. Each container was closed with a transparent lid and put in a growth cabinet (70% RH, 24°C during the light period of 16 h and 20°C during the light period of 8 h). Seedlings were grown on glass beads for 1 week after germination and then stored at 4°C until further use.

### Concentration-dependent inhibitory effects on *J. vulgaris* growth through root inoculation *in vitro*

The two bacterial strains that were isolated from roots of *J. vulgaris* plants, *S. plymuthica* and *P. brassicacearum*, were cultured from the glycerol stocks stored in −80°C freezer. The cultures were incubated in Erlenmeyer flasks containing liquid Tryptic soy broth (TSB) medium on a shaker at 180 r/m and 28°C overnight. The optical density (OD) of the bacterial cultures was measured at a wavelength of 600 nm (OD_600_) and the bacterial suspensions were subsequently spun down in a centrifuge and immediately resuspended in sterilized saline solution (0.9% NaCl) to achieve four OD_600_ values of 0.5, 0.8, 1.0, and 1.34 (the OD_600_ of the overnight culture was 1.34). Seven days after germination of *J. vulgaris* seeds, the roots of seedlings of similar size were immersed in different concentrations of the bacterial solution or in saline solution (control) for 1 min. Following inoculation via dipping, each seedling was immediately transferred to a plate (10 cm × 10 cm × 2 cm) containing 25 ml of 0.5 MS medium (containing 8 g/l agar). In total, there were 38 plates and each plate had one seedling (2 bacterial strains × 4 concentrations × 4 replicate plates + 1 control × 6 replicate plates = 38 plates). The seedlings were allowed to grow for 18 days, and photographs of each seedling were taken at 2-day intervals. Root morphology traits including total root length, primary root length, total lateral root length, and number of lateral roots were determined using the plugin “ObjectJ” (Vischer and Nastase 2022) from ImageJ (the software can be downloaded at https://fiji.sc/). The average total lateral root length was calculated by dividing lateral root length by number of lateral roots. The measurements were obtained from photographs taken on day 18. After 18 days of growth, the seedlings were carefully removed from the plates, and the fresh shoot and root biomass were recorded.

### Concentration-dependent inhibitory effects on *J. vulgaris* growth through root inoculation in soil

Bacteria may develop differently in soil than on agar plates, therefore we also repeated the previous experiment and grew plants in soil. Bacterial solutions of different concentrations of *S. plymuthica* and *P. brassicacearum* (OD_600_ values of 0.5, 0.8, 1.0, and 1.34) and *J. vulgaris* seedlings were prepared as described above. Sterile transparent plastic containers (130 mm height, 30 mm diameter) were used to test the growth of *J. vulgaris* seedlings inoculated with the bacteria but this time the seedlings were planted in soil. Each container was filled with 25 g of sterilized soil, and 2 ml of sterilized MillQ water was added. The soil was collected from a grassland at Lange Dreef in Driebergen, The Netherlands and was sterilized with gamma irradiation (> 25Kgray, Isotron, Ede, The Netherlands). The soil is characterized as a sandy loam holtpodzol with a particle size distribution: 2% <0.002 mm, 11% 0.002–0.063 mm, 84% >0.063 mm, with ∼3% organic matter, 1150 mg/kg N, 61 mg P_2_O_5_ 100/g, 2.4 mmol K/kg, and pH 5.9, and was sieved (0.5 cm mesh size) and homogenized prior to gamma irradiation. The roots of the seedlings were immersed in different concentrations (OD_600_ values of 0.5, 0.8, 1, and 1.34) of bacterial solutions for 30 min and then planted in the soil. Following planting, each plastic container was covered with a transparent lid. In total, there were 46 plants (2 bacterial strains × 4 concentrations × 5 replicates + 1 control × 6 replicates). After 21 days of growth, the plants were harvested and roots were hand-washed to remove the soil. To determine the total root length for each plant, the roots were scanned with an Epson scanner (Epson Perfection V850 Pro) and total root length was determined using the plugin “ObjectJ” from ImageJ. The shoot and root of each seedling were then dried at 60°C for 72 h, and the shoot and root dry mass were measured. The specific root length was calculated as total root length divided by root dry mass.

### Effects of the cell-free supernatants of the two bacterial strains on *J. vulgaris* seedlings

To investigate whether metabolites secreted by the bacteria into the surrounding environment can attribute to the observed negative effects on roots, the effects of cell-free supernatants of the two bacterial strains on *J. vulgaris* seedlings were tested. One week before starting this experiment, *J. vulgaris* seedlings were prepared as described above. Square plates (10 cm × 10 cm × 2 cm) containing 25 ml 0.5 MS medium were prepared. The two bacterial strains were cultured overnight at 28°C in Erlenmeyer flasks containing liquid TSB medium. For inoculation, each of the bacterial cultures was diluted with sterilized TSB medium to an OD measured at a wavelength of 600 nm (OD_600_) equal to 1. Subsequently, the bacterial solutions were spun down again in a centrifuge, and the supernatant was separated. The supernatant of the bacteria (treatment) and fresh TSB medium (as control) were filter-sterilized. Meanwhile, the bacterial pellet was immediately resuspended in sterilized saline solution. Seedlings were inoculated by dipping them for 1 min in the supernatant of one of the bacterial solutions, or in one of the resuspended bacterial solutions, or in sterilized TSB medium (control). In total, there were 25 plates and each contained one seedling [2 bacterial strains × 2 treatments (with or without bacterial cells) × 5 replicates + 1 control (TSB medium) × 5 replicates]. Plates were closed with parafilm and placed vertically in a growth cabinet (70% RH, 24°C during the light period of 16 h and 20°C during the dark period of 8 h). The seedlings were allowed to grow for 12 days, and photographs of each seedling were taken at 2-day intervals. Root morphology traits including total root length, primary root length, total lateral root length, and number of lateral roots were determined using the plugin “ObjectJ” from ImageJ.

### Bacterial effects on *J. vulgaris* plants through root inoculation with or without the ethylene-inhibitor

To examine whether the plant hormone ethylene is involved in the negative effects of the two bacteria on *J. vulgaris*, we used the ethylene inhibitor AVG (Sigma) to inhibit the production of ethylene in plants. Using this method, we hypothesized this would restore the root growth of *J. vulgaris* in the presence of the bacteria. AVG was dissolved in water, filter-sterilized using a GS filter with a 0.22-µm pore diameter, and stored at −20°C prior to use and the concentration of the stock AVG solution was 0.5 mg/l. Half MS medium (containing 8 g/l agar) was prepared and autoclaved to sterilize. When the 0.5 MS medium had cooled down to ~60°C, the stock AVG solution was added to achieve a final concentration of 5 µg/l. We also prepared 0.5 MS medium without AVG addition. Square plates (10 cm × 10 cm × 2 cm) filled with 25 ml of 0.5 MS medium with and without AVG were prepared. Seedlings of *J. vulgaris* and the two bacterial inoculations were prepared as described above. Bacterial cultures were diluted to an OD of 1 at 600 nm (OD_600_). Subsequently, the bacterial solutions were spun down in a centrifuge, and the supernatant was discarded. The bacterial pellet was immediately resuspended in sterilized saline solution. Seedlings were either inoculated by dipping their roots in one of the bacterial inoculations or in sterilized saline solution for 1 min, and then transferred to plates with 0.5 MS medium with or without AVG (each plate contained one seedling). In total, 30 seedlings were used in this experiment: 3 inoculation treatments (2 bacteria + 1 control) × AVG inhibitor (with or without) × 5 replicates. Plates were closed with parafilm and placed vertically in a growth cabinet (70% RH, 24°C during the light period of 16 h and 20°C during the dark period of 8 h). The seedlings were allowed to grow for 12 days, and photographs of each seedling were taken at 2-day intervals. Root morphology traits including total root length, primary root length, lateral root length, and number of lateral roots were determined using the plugin “ObjectJ” from ImageJ. The average total lateral root length was calculated as lateral root length divided by number of lateral roots. The measurements were obtained from photographs taken on day 12.

### Effects of volatiles emitted by bacteria on *J. vulgaris* seed germination

To examine the effects of volatiles emitted by bacteria on seed germination, 10 ml of sterilized Tryptic soy agar (TSA) (for bacterial growth) and of 0.5 MS medium (for seed germination) were each poured into one side of an I-plate (10 cm diameter). The I-plate were divided into two separate compartments allowing volatiles to move between compartments. The two bacterial strains were cultured, and bacterial solutions (OD_600_ = 1) were prepared as described above and we dipped a sterile inoculation loop (1 µl) either in a bacteria solution (OD_600_ = 1, freshly prepared as described above) or in a sterilized saline solution and this was smeared over the TSA side. Each plate was closed with a lid and parafilm and incubated at 28°C overnight. One day later, *J. vulgaris* seeds were surface sterilized as described above. We then used a sterilized toothpick to transfer 25 seeds on the 0.5 MS medium side of each plate. In total, there were 375 seeds in 15 plates (2 bacterial strains × 5 replicated plates × 25 seeds + 1 control × 5 replicated plates × 25 seeds). We recorded the number of freshly germinated seeds on each plate daily for 10 days and calculated the proportion of germination on each plate at day 10.

### Effects of volatiles on *J. vulgaris* seedling growth *in vitro*

To study whether effects of volatiles produced by bacteria on seedling growth of *J. vulgaris* are concentration-dependent, a similar procedure with the I-plate that were mentioned above were used. One week before initiating the experiment, *J. vulgaris* seeds were surface sterilized and germinated on 0.5 MS medium as described above. Sterilized TSA and 0.5 MS medium were prepared and poured in one of the sides of each split Petri dish, following the method described above. Bacteria were cultured and bacterial solutions (OD_600_ = 1) were prepared as described above. Immediately afterward, we inoculated one of the bacteria or sterilized saline solution (control) on the TSA medium by pipetting (i) 20 µl (1 spot), (ii) 3 times 20 µl (3 spots), or (iii) 5 times 20 µl (5 spots). The plates were closed with parafilm and put in an incubator at 28°C overnight. One day after inoculation, we transferred four *J. vulgaris* seedlings onto the 0.5 MS medium side of each plate. The plates were sealed with parafilm, and photographs at fixed height were taken of each seedling. In total, there were 54 plates and each plate had four seedlings (2 bacterial strains × 3 concentration treatments × 6 replicated plates + 1 control × 3 concentration treatments × 6 replicated plates). The plates were then placed vertically in a growth cabinet (70% RH, 24°C during 16 h of light and 20°C during 8 h of darkness). We traced root growth of each seedling by taking photographs of each seedling at fixed height. Ten days after placing the seedlings in the plates, the experiment was ended. Total root length of the four seedlings per plate at day 10 was determined using the plugin “ObjectJ” from ImageJ. The total shoot and roots of all seedlings per plate were dried at 60°C for 72 h, and the combined shoot and root dry mass of all seedlings per plate were measured.

### Effects of volatiles on seedling growth of *J. vulgaris* in soil

To examine whether bacterial volatiles have an effect on *J. vulgaris* grown in soil, we utilized a pot-in-jar system with some modifications based on Martín-Sánchez et al. (2020). One week before the experiment, *J. vulgaris* seeds were surface sterilized and germinated on plates with 0.5 MS solid medium as described above. Six days after germination, we prepared sterilized TSA and TSB medium. Bacteria were cultured and bacterial solutions (OD_600_ = 1) were prepared as described above. In each sterilized plant tissue culture container (53 mm diameter, 100 mm height), we added 20 ml of sterilized TSA medium. Once the TSA medium was solid, we pipetted either 50 µl of one of the bacteria solutions or 50 µl of sterilized TSB medium onto the TSA medium in each container. We closed and sealed all containers with a lid and then incubated them at 28°C for 24 h. Seven days after germination, we made twelve 2 mm diameter holes in the bottom of each 60 ml measuring cup and placed a filter paper (4 cm diameter) at the bottom. Each measuring cup was filled with 50 g of gamma-radiated sterilized soil, and the soil was the same soil as described above. One seedling was transferred into each cup. Immediately afterwards, in a sterile flow cabinet, we randomly selected a measuring cup with a grown seedling and placed it above the bacteria culture container. The connection between the measuring cup and the bacteria culture container was sealed with parafilm. In total, there were 60 plants [3 treatments (2 bacteria treatments + 1 control) × 20 replicates]. Subsequently, they were placed in a growth cabinet (70% RH, 24°C during 16 h of light and 20°C during 8 h of darkness). The plants were watered when needed and were harvested 28 days after planting the seedlings. The shoot and roots of each plant were dried at 60°C, and the shoot and root dry mass were recorded.

### Effects of the two bacterial strains on seedling growth of *J. vulgaris* and nine co-occurring plant species *in vitro*

One week before this experiment, sterilized seeds of *J. vulgaris* and nine other co-occurring plant species were germinated on sterilized glass beads as described above. Square plates with 0.5 MS growth medium were prepared. The two bacterial strains were cultured overnight at 28°C in Erlenmeyer flasks containing liquid TSB medium. For inoculation, the cultures were diluted to an OD of 1 at 600 nm (OD_600_). Subsequently, the bacterial solutions were spun down in a centrifuge, and the supernatant was separated. The bacterial pellet was immediately resuspended in sterilized saline solution. Seedlings were inoculated by dipping them for 1 min either in one of the bacterial solutions or in sterilized saline solution. Immediately afterwards, each seedling was transferred on one square plate with 0.5 MS growth medium. In total, there were 150 plates with one seedling (10 plant species × 2 bacterial strains × 5 replicates + 1 control × 10 plant species × 5 replicates). Plates were closed with parafilm and placed vertically in a growth cabinet (70% RH, 24°C during 16 h of light and 20°C during 8 h of darkness). Photographs of each seedling were taken at 2-day intervals. Root morphology traits including primary root length and total root length were determined using the plugin “ObjectJ” from ImageJ. The seedlings were allowed to grow for 12 days after which fresh shoot and root biomass of each seedling were measured.

### Volatile analysis by Headspace-GC–MS

The two bacteria strains, *S. plymuthica* and *P. brassicacearum*, were cultured and prepared (OD _600_ = 1) as described above. Headspace vials (20 ml, 75.5 mm × 22.5 mm) with screw top, clear glass with silver magnetic screw caps with white silicone/Red PTFE septa were used for the bacteria incubation and volatile collection. Headspace vials and their caps, and TSA medium were sterilized by autoclaving. Hereafter, each vial was first filled with 500 µl TSA and placed the top of vial on the screw cap (a slope around 15°) on the floor of the UV cabinet. After the TSA turned solid on one side, we immediately added another 500 µl TSA to each vial and we let TSA become solid on the opposite side of each vial. This increased the surface area of medium for bacteria growth. We then added twice of 50 µl of bacterial inoculum or TSB liquid medium (control) to inoculate the TSA medium on both sides of a vial, and hence each vial received in total 100 µl bacterial inoculum or TSB liquid medium (control). Headspace vials were then closed by screw caps. As the composition and concentration of bacterial volatiles also depends on the incubation time, we incubated samples for 2 and 6 days in an incubator at 28°C. In total, there were 18 headspace vials [3 treatments (2 bacterial inoculum + 1 control) × 3 replicates × 2 time intervals]. Volatiles measured using GC–MS (Agilent, Folsom, CA, USA). The headspace parameters including incubation temperature and incubation time were carefully configured to facilitate the extraction of volatiles released from the bacteria strains. Each vial was incubated at 35°C for 40 min at 500 r/m to promote the release of volatiles, then 500 µl was sampled and injected into the gas chromatograph. GC–MS measurements were performed using a 7890A gas chromatograph equipped with a 7693 automatic sampler and a 5975C single-quadrupole mass detector (Agilent). Volatile compounds were separated on a DB-5 column: 30 m × 0.25 mm, 0.25 µm film (J&W Scientific, Folsom, CS, USA), using Helium (99.9% purity) as a carrier gas at a flow rate of 1.6 ml/min. The injections were done using a splitless mode (50 ml/min at 2 min). The oven temperature was programmed to start at 35°C, then held for 6 min, increased at steps of 10°C/min to 130°C, held for 1 min and then further increased to 175°C at steps of 20°C/min, which was held for 1 min. The ionization energy on EI mode was 70 eV, and peak identification was made by comparing the ion spectra obtained from the samples versus ion spectra in the NIST library (version 2014).

### Genome sequencing of the two bacteria isolates

The two bacterial isolates were cultured overnight at 28°C in an Erlenmeyer with liquid TSA medium for DNA extraction. The DNA extraction was performed using DNeasy® Blood and Tissue Kit (QIAGEN, Hilden, Germany) according to the manufacturer’s instructions. The genome sequence of *S. plymuthica* and *P. brassicacearum* was determined based on paired-end sequencing using the Illumina GAIIx platform. The pair-end reads were de novo assembled using the CLC Genomic Workbench version 4.7.2 (CLC bio). Genes were identified using Prodigal as part of the genome annotation pipeline at Oak Ridge National Laboratory (ORNL), Oak Ridge, TN, USA, followed by a round of manual curation using the JGI GenPRIMP pipeline.

### Data analysis

The effects of bacterial inocula concentration (treated as numeric variables) on various root traits, shoot and root biomass of *J. vulgaris* were examined using linear regression models for each bacterial strain, respectively. The control treatment was not included in the linear regression models. One-way ANOVA was then employed to examine the effects inoculation treatments including the control (treated as factor variables, nine levels), followed by a Tukey *post hoc* test, to compare the difference between bacterial inoculation and the control treatment. To test the effects of cell free supernatant of the two bacteria, one-way ANOVA was used to assess the effects of inoculation treatments (control, two bacterial solutions, and two supernatants) on root traits. To examine whether the ethylene-inhibitor (AVG) will restore the effects of bacterial inocula, a two-way ANOVA was used to examine effects of inoculation treatments, the presence of the ethylene-inhibitor and its interaction on root traits. A Tukey *post hoc* test was then used to compare differences among treatments.

The effects of bacterial volatiles on seed germination proportion, shoot and root dry mass, and total root length of *J. vulgaris* plants were examined using one-way ANOVA. The effects of bacterial inoculation, concentration of bacterial inocula, and its interaction on shoot and root dry mass and total root length of seedlings per plate (four seedlings together) were examined using two-way ANOVA. One-way ANOVA was also used to examine the effect of the incubation period on the concentration of volatile compounds. A Tukey *post hoc* test was then used to compare the differences among treatments. To examine whether these two bacteria also affect other plant species, the effects of bacterial inoculation and identity of plant species on primary root length, total root length, fresh shoot, and root biomass of *J. vulgaris* and nine other co-occurring plant species were examined using two way-ANOVA. A one way-ANOVA was then used for each plant species separately, followed by a Dunnett *post hoc* test to compare the difference between bacterial inoculation and the control treatment. All statistical analyses were performed in R, version 4.2.2 (R Core Team 2022), with checks for homogeneity of variance and normality of residuals. Primary root length, total root length, and fresh root biomass were log-transformed to fulfill requirements of normality.

## Results

### Concentration-dependent inhibitory effects on *J. vulgaris* growth through root inoculation

**Figure 1.**
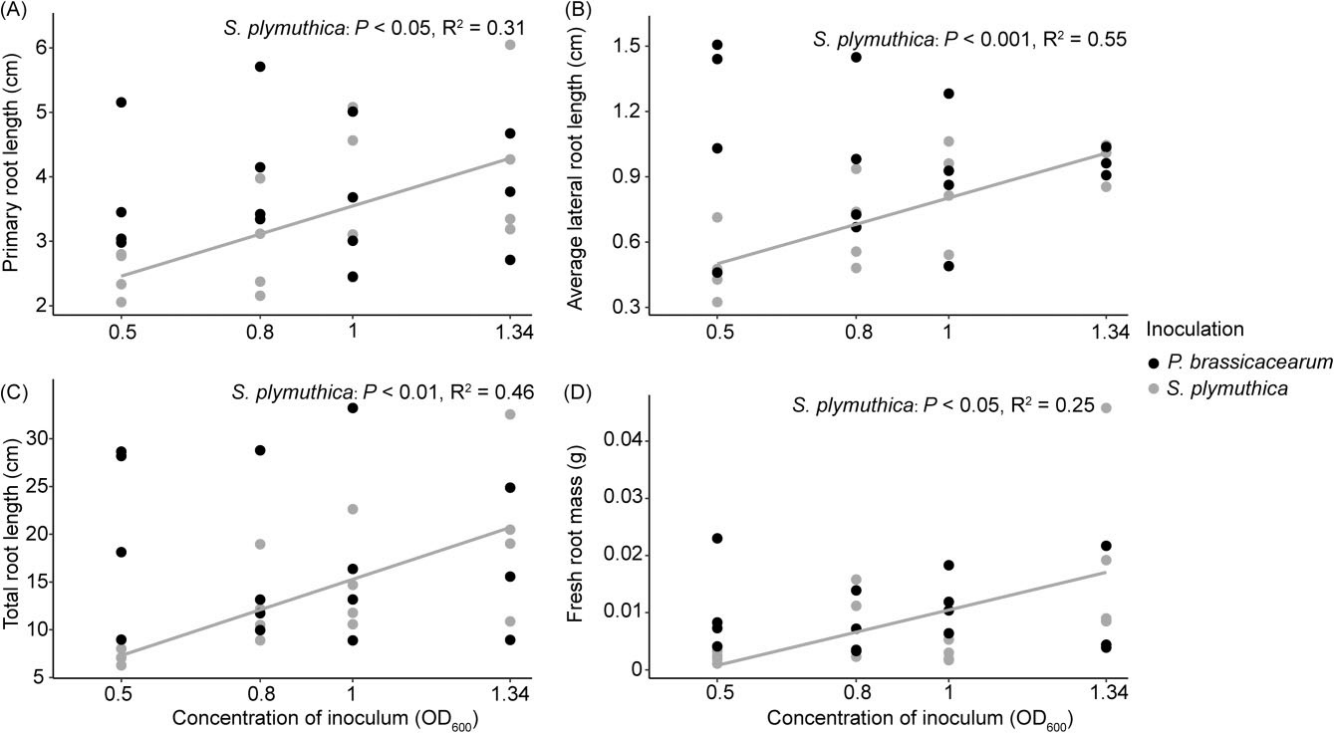
Relationship between the concentration of the bacterial inoculum and primary root length (A), average lateral root length (B), total root length (C), and fresh root mass (D) of *J. vulgaris in vitro*. In (A, B, C, and D), *R*^2^ and *P*-values from a linear regression analysis for *S. plymuthica* are also presented. There were no significant relations for *P. brassicacearum*.

**Figure 2.**
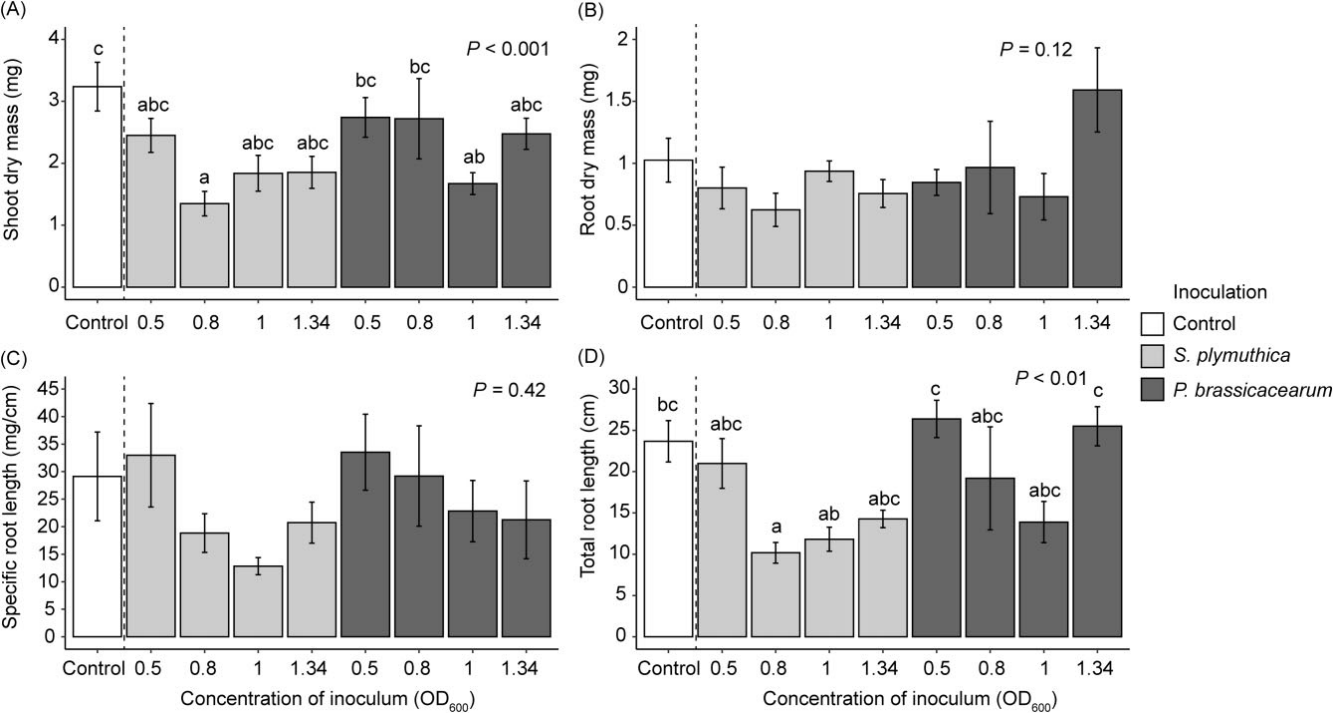
Mean (± SE) shoot dry mass (A), root dry mass (B), specific root length (C), and total root length (D) of *J. vulgaris* in the presence of one of the two bacterial inocula at varying concentrations in soil. In (A and D), letters indicate significant differences between the inoculation treatments (*P* < .05) based on a Tukey *post hoc* test and bars with identical letters are not significantly different. In (B and C) there were no significant differences between the inoculation treatments. *P-*values of an one-way ANOVA are also presented.

**Figure 3.**
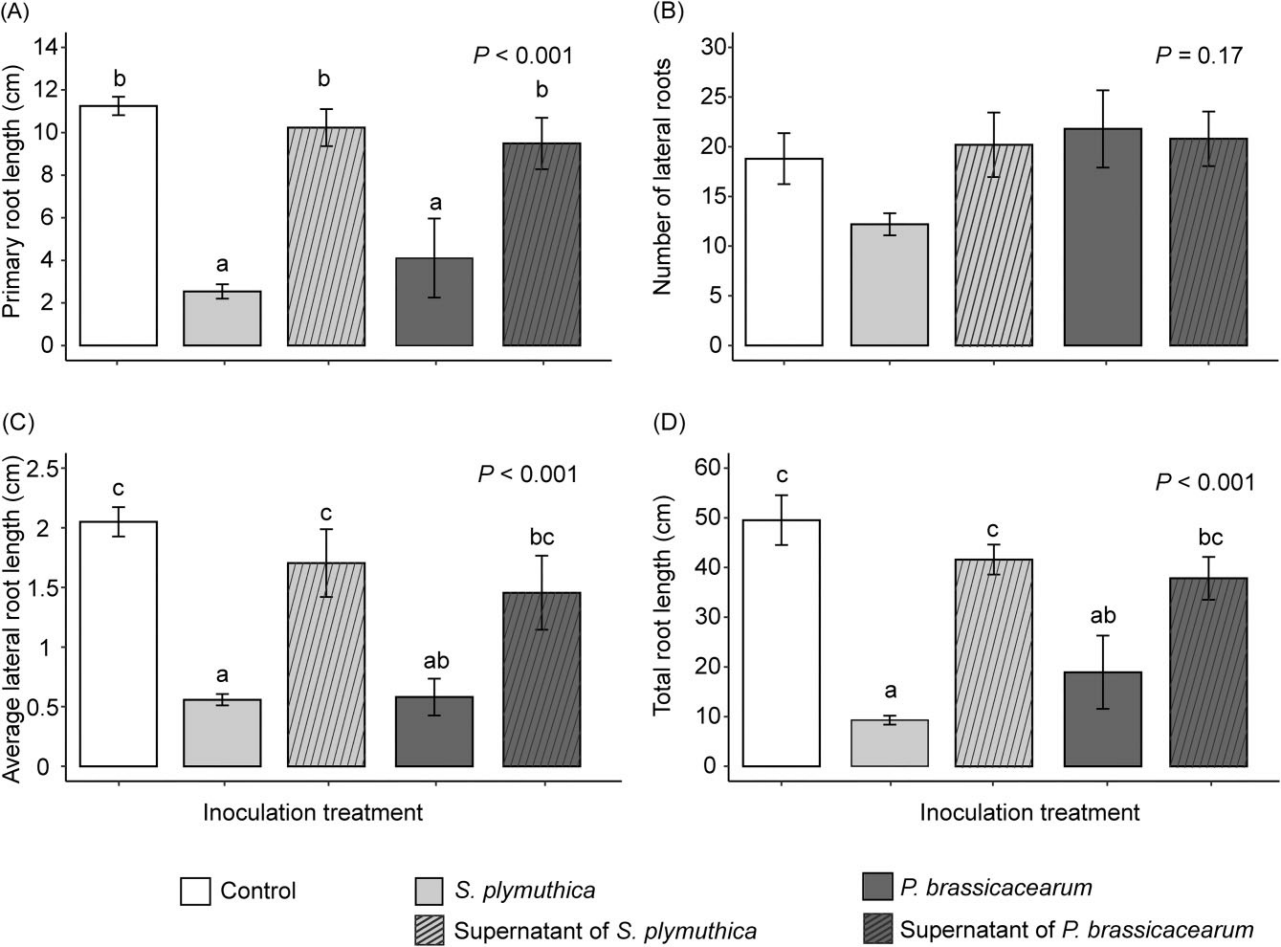
Mean (± SE) primary root length (A), number of lateral roots (B), average lateral root length (C), and total root length (D) of *J. vulgaris* seedlings with bacterial cells or supernatant of one of the two bacteria or no inoculation (control) in *vitro*. In (A, C, and D), letters indicate significant differences between the inoculation treatments (*P* < .05) based on a Tukey *post hoc* test and bars with identical letters are not significantly different. In (B), there were no significant differences between inoculation treatments. *P-*values of an one-way ANOVA are also presented.

**Figure 4.**
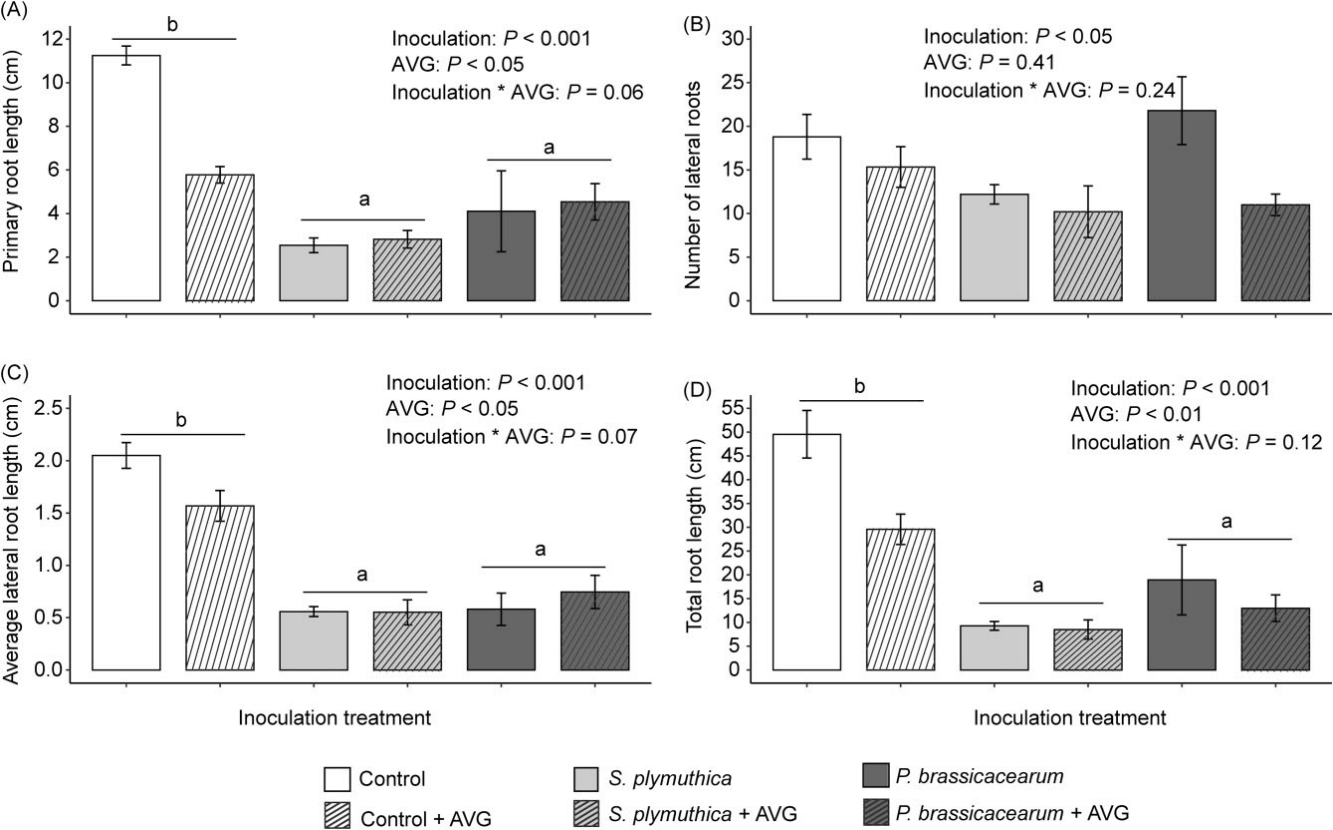
Mean (± SE) primary root length (A), number of lateral roots (B), average lateral root length (C), and total root length (D) of *J. vulgaris* seedlings 10 days after inoculation with one of the two bacterial inocula or no inoculation (control) with or without the ethylene inhibitor (AVG). In (A, C, and D), letters above the horizontal line indicate significant differences between inoculation treatments (*P* < .05) based on a Tukey *post hoc* test and bars with identical letters are not significantly different. In (B), there were no significant differences between inocula. *P*-values of a two-way ANOVA are also presented.

Bacterial inoculation significantly reduced total root length of *J. vulgaris* seedlings, primarily through reducing the length of the primary root (Fig. S1A). The inhibitory effects were also observed for fresh root biomass when the inoculation concentration of *S. plymuthica* was 0.5 at OD_600_ (Fig. S1F). However, bacterial inoculation had no effect on number of lateral roots, fresh shoot biomass, and the concentration of leaf chlorophyll and carotenoids (Table S1, Fig. S1B, E, G, and H). The concentration-dependent effect was observed only for *S. plymuthica*, where an increasing bacterial concentration led to a reduction in the negative effects on root growth *in vitro* (Fig. 1, Table S1). When *J. vulgaris* plants were grown in soil, *S. plymuthica* through root inoculation only significantly reduced shoot dry mass and total root length of *J. vulgaris* plants when the inoculation concentration was 0.8 at OD_600_, compared to the control (Fig. 2). Inoculation with *P. brassicacearum* only led to significant reduction in shoot dry mass when inoculation concentration was 1 at OD_600_ compared to the control treatment (Fig. 2). There was no concentration-dependent pattern for both bacteria when plants were grown in soil (Table S2). The supernatant of the bacterial solution had no effect on root morphological traits (Fig. 3, Table S3). There was no significant interaction effect between bacterial inoculation and the presence of the ethylene inhibitor AVG on root traits, indicating that the ethylene inhibitor AVG did not restore the negative effects of bacteria on root growth of *J. vulgaris* (Table S4). Instead, the ethylene inhibitor itself exhibited negative effects on root growth (Fig. 4, Table S4).

### Volatile effects of bacteria on seed germination and seedling performance of *J. vulgaris*

**Figure 5.**
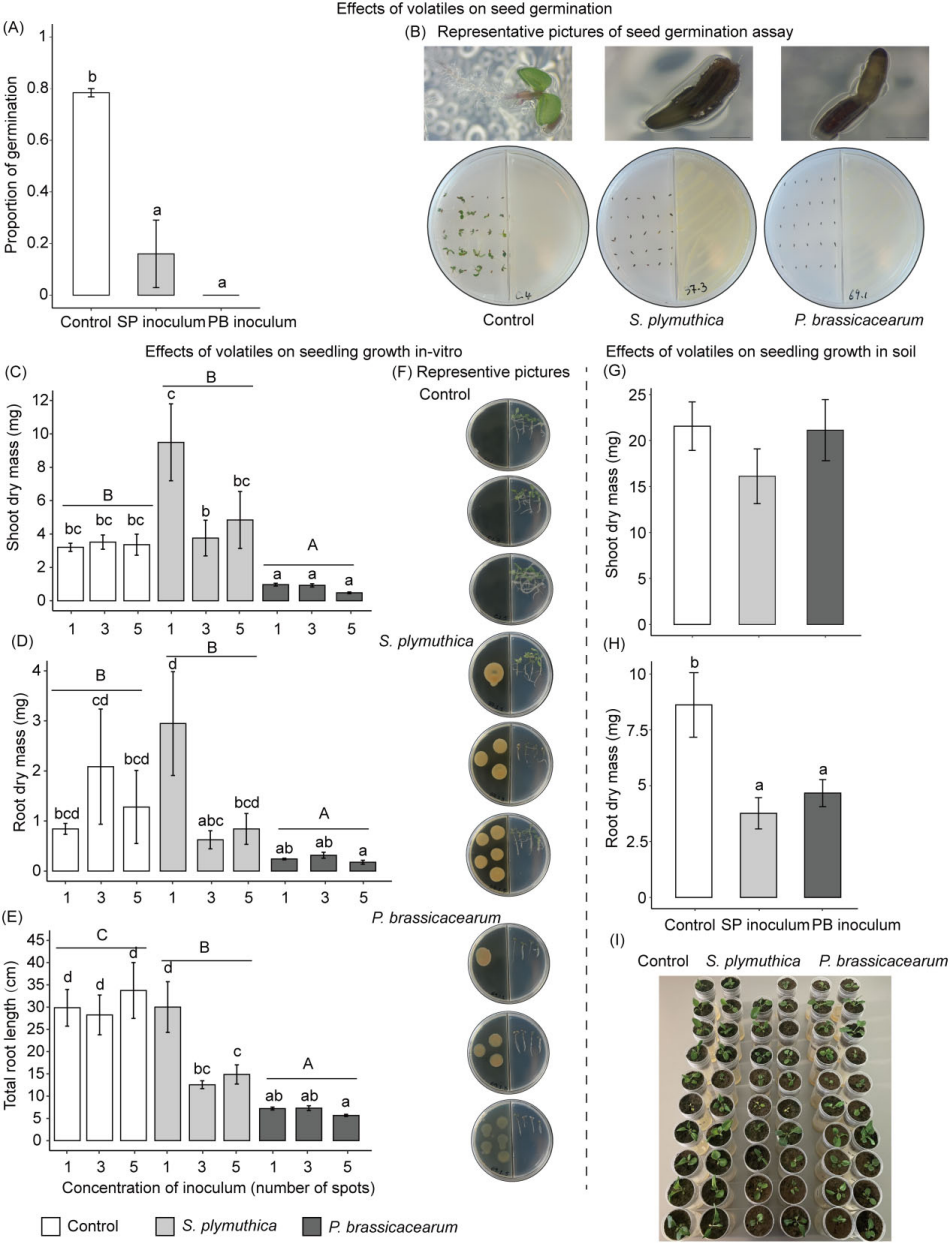
Mean (± SE) proportion of germination of *J. vulgaris* seeds 10 days after the start of the experiment in presence or absence of volatiles emitted by one of the two bacterial strains (A), representative pictures of the seed germination assay (B), mean (± SE) shoot dry mass (C), root dry mass (D), total root length (E), and representative pictures (F) of *J. vulgaris* seedlings per plate 10 days after placing them in plates in the presence of bacterial volatiles with 20 (one spot), 60 (three spots), and 100 µl (five spots) of bacterial inoculum, and shoot (G) and root dry mass (H) and a picture (I) of *J. vulgaris* plants 28 days after planting in soil after exposure to volatiles of one of the two root-associated bacteria. In (A, C, D, E, and H), letters above bars indicate significant differences between treatments (*P* < .05) based on a Tukey *post hoc* test and bars with identical letters are not significantly different. In (G), there were no significant differences between treatments. In (C, D, and E), capital letters above the horizontal line indicate significant differences between bacterial inocula (*P* < .05) based on a Tukey *post hoc* test.

Bacterial volatiles significantly inhibited seed germination of *J. vulgaris* (Fig. 5A and B). The average proportion of germinated seeds was 0.78 (SE = 0.02) in the control treatment without bacterial volatiles. In presence of volatiles from *S. plymuthica* and *P. brassicacearum*, the proportion of germinated seeds was reduced to 0.16 (SE = 0.13) and 0, respectively (Fig. 5A). Volatiles from *S. plymuthica* significantly reduced total root length per plate but had no influence on shoot and root dry mass of *J. vulgaris* seedlings per plate *in vitro* (Fig. 5C–E). Volatiles from *P. brassicacearum* significantly reduced shoot and root dry mass, and total root length of *J. vulgaris* seedlings per plate *in vitro* (Fig. 5C–E). The concentration effect was not the same for all inoculation treatments resulting in a significant interaction between inoculation treatment and concentration (Table S5). In particular, volatiles from *S. plymuthica* only led to a reduction in total root length at high concentrations (three and five spots of bacterial inoculum) (Fig. 5E). In soil, exposure to volatiles from both bacterial strains significantly reduced root dry mass of *J. vulgaris* (F_2, 55_ = 6.58, *P* < .01), while it had no effect on shoot dry mass (Fig. 5G–I, F_2, 56_ = 1.01, *P* = .37).

### Volatile profiles of the two bacteria from HS-GC–MS

**Figure 6.**
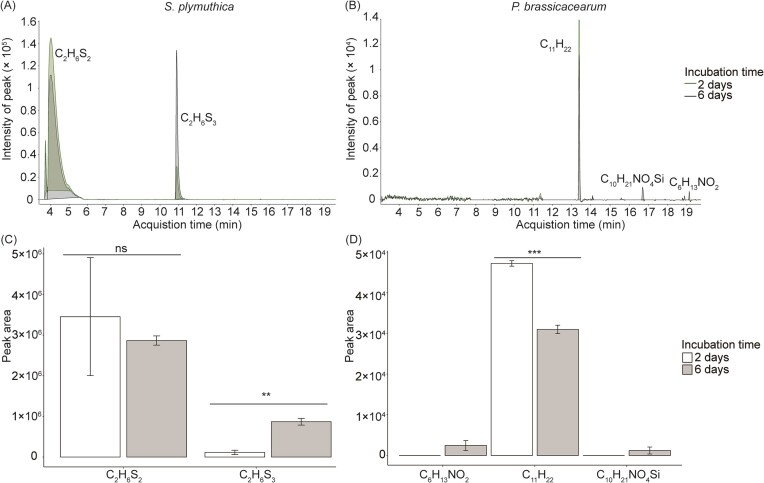
GC–MS chromatogram of volatiles of *S. plymuthica* (A) and *P. brassicacearum* (B), and mean (± SE) peak area of the compounds after 2 and 6 days of incubation of the bacteria (C and D). In (A) and (B) bacteria was incubated for 2 and 6 days in the HS-GC–MS vials, respectively (one out of three samples is presented). Structural formulas of compounds: C_2_H_6_S_2_ = dimethyl disulfide, C_2_H_6_S_3_ = dimethyl trisulfide, C_11_H_22_ = cyclopropane-octyl, C_10_H_21_NO_4_Si = methyl 2-hydroxy-4-methyl-4-nitroso-pentanoate, and C_6_H_13_NO_2_ = acetic acid. In (C) and (D), **, *** indicates significant differences at *P* < .01 or .001, respectively based on a Tukey *post hoc* test.

Distinct volatile profiles were identified from the two bacterial strains, *S. plymuthica* and *P. brassicacearum*, using HS-GC–MS. (Fig. 6A and B, Table S6). Specifically, volatiles emitted by *S. plymuthica* were found to contain DMDS and dimethyl trisulfide (DMTS). Volatiles emitted by *P. brassicacearum* contained cyclopropane-octyl, methyl 2-hydroxy-4-methyl-4-nitroso-pentanoate and acetic acid (Table S6). Furthermore, the concentration of DMDS did not differ, but the concentration of DMTS was higher after 6 days of incubation compared to 2 days for *S. plymuthica* (Fig. 6C). Regarding volatiles emitted by *P. brassicacearum*, cyclopropane-octyl was consistently detected in both incubation periods, while methyl 2-hydroxy-4-methyl-4-nitroso-pentanoate and acetic acid were only detected in samples incubated for 6 days (Fig. 6D).

### Effects of the two bacteria on growth of *J. vulgaris* and other co-occurring plant species

**Figure 7.**
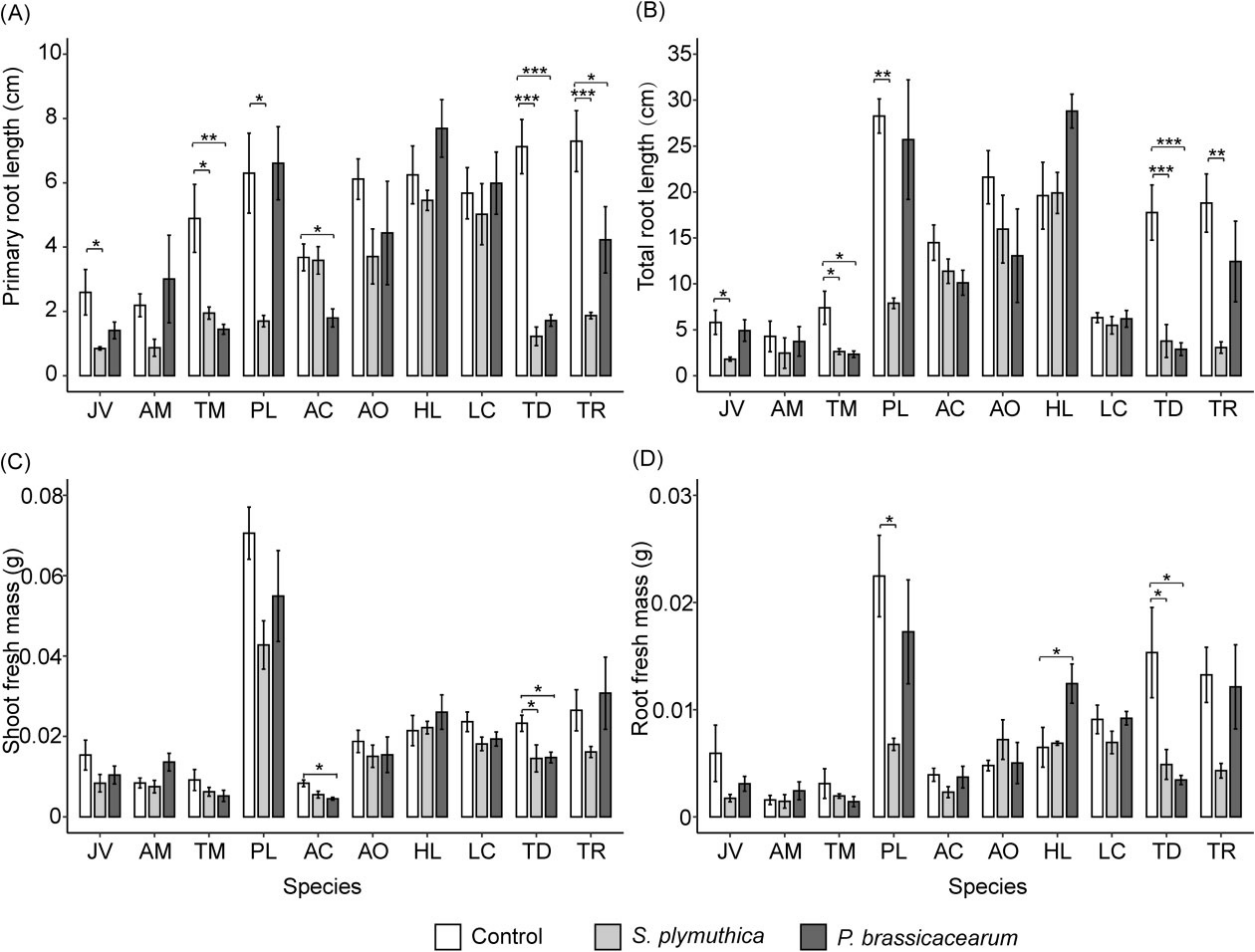
Mean (± SE) primary root length (A), total root length (B), shoot (C), and root fresh mass (D) of *J. vulgaris* and nine other plant species in the presence and absence of one of the two bacteria *in vitro*. In (A, B, C, and D) asterisks indicate significant differences between bacterial inoculum and control treatment based on a Dunnett *post hoc* test of a one-way ANOVA for each plant species. *, **, and *** indicates significant differences at *P* < .05, .01, or .001, respectively. Abbreviations of species: JV = *Jacobaea vulgaris*, AM = *Achillea millefolium*, TM = *Tripleurospermum maritimum*, PL = *Plantago lanceolata*, AC = *Agrostis capillaris*, AO = *Anthoxanthum odoratum*, HL = *Holcus lanatus*, LC = *Lotus corniculatus*, TD = *Trifolium dubium*, and TR = *Trifolium repens*.

The effects of bacterial inoculation on the length of the primary root and total root length, varied significantly depending on the identity of the plant species (Fig. 7A and B, Table S7). Inoculation with *S. plymuthica* significantly reduced primary root length and total root length in *J. vulgaris*, as well as in two other forbs (*T. maritimum* and *P. lanceolata*), and two legumes (*T. dubium* and *T. repens*) (Fig. 7A and B). Similarly, inoculation with *P. brassicacearum* significantly reduced primary root length in *T. maritimum, A. capillaris, T. dubium*, and *T. repens*, but this effect is not significant for *J. vulgaris* (Fig. 7A and B). Root biomass of *P. lanceolata* and *T. dubium* were significantly reduced after inoculation with *S. plymuthica*. Inoculation with *P. brassicacearum* significantly reduced shoot biomass of *A. capillaris* and *T. dubium*, but increased root biomass of *H. lanatus* (Fig. 7C and D).

### Genome properties

The genome of *S. plymuthica* consists of 5 651 564 bases and includes 6 rRNA sequences, 86 tRNA sequences, 1 tmRNA sequence, and 5164 coding DNA sequences (CDS) that encode proteins (Table S8). The genome of *P. brassicacearum* comprises a total of 6 996 581 bases and features 5950 CDS, 4 rRNA sequences, 68 tRNA sequences, #tmRNA (Table S8). The hcnABC operon genes were identified in *P. brassicacearum* based on the annotation.

## Discussion

The central focus of this study was to examine the mechanisms underlying the negative effects of two bacteria, *S. plymuthica* and *P. brassicacearum* isolated from roots of *J. vulgaris* on plant performance. We investigated the effects of bacterial strains on the performance of *J. vulgaris* via root inoculation and via volatiles at various concentrations of the bacterial solution. Additionally, we explored whether the two root-associated bacteria, isolated from *J. vulgaris*, exhibit host specificity. This study reveals four main findings. First, both bacterial strains negatively affected *J. vulgaris* plants through root inoculation, while only the inoculum of *S. plymuthica* exhibited a concentration-dependent pattern *in vitro*. Second, the negative influence of the bacteria on performance of *J. vulgaris* through root inoculation was attributed to the activity of bacterial cells rather than the cell-free supernatants in the suspensions. Third, volatiles produced by the two bacteria inhibited seed germination and plant growth and distinct volatiles were observed for the two bacterial strains. Lastly, the effects of the two root-associated bacteria on plant growth were not specific to *J. vulgaris* and these effects varied among a range different plant species belonging to three different functional groups. Based on this study, we can conclude that the negative effects of two root-associated bacteria on *J. vulgaris* growth are mediated through root inoculation with bacterial cells rather than through the metabolites produced by these bacteria in the supernatant alone, and through volatiles produced by the bacteria.

Previous studies have reported that when root-associated bacteria impact plants through the production of metabolites, these effects are often concentration-dependent (Shantharaj et al. 2021, He et al. 2023). In our study, only *S. plymuthica* exhibited a concentration-dependent effects on *J. vulgaris in vitro*. Furthermore, the cell-free supernatant of both bacteria did not influence *J. vulgaris* performance indicating the negative effects were likely due to direct interaction between bacteria and plants rather than through the production of nonvolatile metabolites. Regarding to the concentration-dependent effect of *S. plymuthica*, the patterns that were observed *in vitro* and in soil were opposite. When bacterial inoculum was at the lowest concentration (OD_600_ = 0.5), plant performance was lowest on agar plates, while this pattern was reversed when plants were grown in soil. On agar plates, although the initial bacterial concentrations in the inoculum varied, we expected that bacteria grew rapidly, utilizing the available carbon (C) released by roots. Subsequently, this may have led to competition between bacteria and the plant at all concentrations and this may explain the overall decline in root growth (Kuzyakov and Xu 2013). However, to our surprise, the negative effects of *S. plymuthica* decreased with an increasing bacterial concentration. One possible explanation is that at higher concentrations, the bacteria may exhibit a shift in their behavior or metabolism (referred to as quorum-sensing mechanism) (Mukherjee and Bassler 2019), leading to a reduction in the negative effects on root growth. This shift could result in a less deleterious interaction between the bacteria and the plant. Further investigations could focus on understanding the specific mechanisms underlying this concentration-dependent effect, such as examining the changes in gene expression in both the bacteria and the plant upon interaction at varying concentrations. When plants were grown in soil, negative effects of the two bacteria on plant growth disappeared at the lowest concentration of the inoculum. This may be because bacteria multiply more slowly in soil, or because bacteria did not survive in soil at the lowest concentrations. This could also explain why the negative effects were more pronounced in growth medium than in soil. Future studies should determine how rapidly the two bacterial populations increase in soil after inoculation.

In contrast to our expectation, there were no significant interaction effects between bacterial inoculation and the presence of the ethylene inhibitor (AVG). Notably, the presence of the ethylene inhibitor itself significantly reduced root growth of *J. vulgaris*. AVG is known to inhibit the synthesis of ethylene by plants (Adams and Yang 1981, Yang and Hoffman 1984). A previous study has found that the presence of AVG suppressed the development of short and thick roots in *V. sativa* plants that are induced by Rhizobium bacteria (Zaat et al. 1989). In our study, the presence of AVG did not restore the root phenotype induced by bacterial cells, but instead, reduced root growth. One possible explanation is that the concentration of AVG used in the experiment was too high. Based on these results we cannot conclude that the inhibitory effects on root growth by the two bacteria are caused by the production of ethylene in plants. To confirm whether bacterial inoculation reduces root growth through the induction of the ethylene pathway, gene expression in plants needs to be examined.

Volatiles from *S. plymuthica* reduced seed germination by 62%, and volatiles from *P. brassicacearum* completely inhibited seed germination. Furthermore, volatiles from *P. brassicacearum* consistently suppressed seedling growth, whereas volatiles from *S. plymuthica* had no effect on seedling growth at the lowest concentration but inhibitory effects appeared at higher concentrations. Many *Pseudomonas* strains have been found to release HCN, which is extremely toxic for living organisms (Blom et al. 2011b, Bailly et al. 2014, Ossowicki et al. 2017). In our study, *P. brassicacearum* has been found to produce HCN as well (see preliminary results in Fig. S2). Therefore, the consistent inhibitory effects of *P. brassicacearum* volatiles on seed germination may be attributed to the release of HCN. Although three HCN-related genes (hcnA, hcnB, and hcnC) were found from the genome annotation of this bacterial strain, we did not detect HCN using HS-GC–MS (Audrain et al. 2015). The reason for that may be the context-dependent production of this toxic metabolite in response to environmental factors, potentially the oxygen level, volatile compounds released by seeds or quorum-sensing mechanisms since the collection of volatiles was performed for a pure culture of bacteria. The regulation of HCN production was studied in *Pseudomonas aeruginosa* by Blumer and Haas (2000) and has been attributed to ANR gene related to sensing the oxygen levels and gacS/gacA global regulator responding to various factors. To our knowledge, the regulation of HCN production has not been studied in environmental bacteria including *P. brassicacearum*. Future studies are required to measure the detailed volatile profiles using preconcentration methods such as SPME, in interactions with plant and seeds (Sharifi and Ryu 2018, Sharifi et al. 2022). Previous studies have reported that the effects of bacterial volatiles on seedling growth were highly dependent on the amount of inoculum used (Blom et al. 2011a). Some studies have found that *S. plymuthica* can promote plant growth, while other evidence suggests that volatiles emitted by *S. plymuthica* have negative effects on plant growth (Velázquez-Becerra et al. 2011, del Carmen Orozco-Mosqueda et al. 2013, Zamioudis et al. 2015). The reported volatiles emitted by *S. plymuthica* include carbon dioxide, ammonia, dimethyl trisulfide (DMTS) and sodorifen (Mai 2018). Notably, we observed that when the amount of inoculum of *S. plymuthica* was the lowest (one spot), plants exhibited enhanced growth particularly of shoot dry mass, although this did not differ significantly from the control. One possible explanation is that plants utilized the carbon dioxide emitted by *S. plymuthica*. We speculate that at higher concentrations, the accumulation of dimethyl compounds (DMDS and DMTS) may have led to negative effects on *J. vulgaris*. In our HS-GC–MS detection, we did observe higher concentration of DMTS with a longer incubation period. Further studies are needed to examine the dose-dependent effects of specific (synthetic) compounds present in the volatiles released by *S. plymuthica* on plant growth.

The negative effects of the two bacteria were not specific to *J. vulgaris*, but also affected some of the other plant species tested. One primary challenge in weed control using microbes is to identify microbes that suppress weeds without negatively impacting nonweedy species (Trognitz et al. 2016). Our study also shows that grasses, overall, appeared to be less affected by bacterial inoculation. This may be because grasses often have fibrous roots, which made them less susceptible to the impact of bacterial cells (Fry et al. 2018). Interestingly, we observed that *P. brassicacearum* caused an increase in root biomass of the grass *H. lanatus*, and other studies have shown that *H. lanatus* grows well in the soil conditioned by *J. vulgaris* (Bezemer et al. 2018, Hannula et al. 2021, Steinauer et al. 2023). This suggests that *H. lanatus* may be able to utilize extracellular enzymes or other compounds released by *P. brassicacearum* but further studies are needed to test this hypothesis.

## Conclusion

Our study demonstrates that both bacterial cells and volatiles from two root-associated bacteria negatively affect root growth of *J. vulgaris*. However, these effects are not specific to *J. vulgaris* and both bacteria also have negative effects on the growth of other plant species. *J. vulgaris* is an unwanted outbreak species in Europe and an invasive species in other continents. Even though the bacteria have negative effects on the growth of *J. vulgaris*, their negative effects on other plant species hampers their potential use as biocontrol agents.

## Supplementary Material

fiae116_Supplemental_File
