# Supplementary material for: Dissecting negative effects of two root-associated bacteria on the growth of an invasive weed
Source: FEMS Microbiol Ecol. 2024 Aug 22;100(10):fiae116. doi: 10.1093/femsec/fiae116 (PMC11879465; doi:10.1093/femsec/fiae116)

**Concentration-dependent inhibitory effects on *J. vulgaris* growth through root inoculation *in vitro***

**Chlorophyll and carotenoid content**

To determine the effects of the bacteria on plant health, chlorophyll and carotenoid content of the leaf were measured following a modified protocol of Kshetrimayum et al. (2018). The original publication of that describes the quantification formula was from Lichtenthaler (1987). Approximately 0.05 gram of fresh leaf material from each seedling was cut and placed, along with a metal ball, into a 2 ml Eppendorf tube. The weight of fresh leaf material used was recorded as well. Then, 1 ml of 95% Ethanol was added to each Eppendorf tube. The tubes were shaken for 1 minute at 1800 rpm using the TissueLyser II. After shaking, the Eppendorf tubes were centrifuged for 2 minutes at 13,200 rpm (maximum speed) at room temperature and 0.5 ml of the supernatant was mixed with 4.5ml of 95% Ethanol in a clean Falcon tube (15 ml). Then 200 µL of each mixture was pipetted into a 96-well plate. One well included 95% ethanol as the control treatment. The optical density (OD) was then measured at 649 nm, 664 nm and 470 nm using a Tecan Spark 10M Microplate Reader (Männedorf, Switzerland). Chlorophyll and Carotenoid contents were calculated using the following standard formulas: Chlorophyll a = 13.36*A_664_ – 5.19*A_649_; Chlorophyll b = 27.43*A_649_ – 8.12*A_664_; Carotenoids = (1000*A_470_ –2.13*Chlorophyll a – 97.63* Chlorophyll b)/209 (Kshetrimayum *et al.* 2018). Chlorophyll and Carotenoid contents were then calculated per gram of fresh leaf material.**Table S1** Results of linear regression models testing the relationship between concentration of bacterial inoculation and root morphology traits, fresh shoot and root biomass, and leaf chlorophyll and carotenoid content of *J. vulgaris* seedlings through root inoculation *in vitro*. Presented are slopes, *P* values and adjusted R^2^ from linear regression models. *, **, or *** indicate significant difference at *P* < 0.05, 0.01 or 0.001.

|  | *S. plymuthica* inoculum | | | *P. brassicacearum* inoculum | | |
| --- | --- | --- | --- | --- | --- | --- |
|  | Slope | P value | R^2^ | Slope | P value | R^2^ |
| Primary root length | 2.18 | 0.01* | 0.31 | -0.10 | 0.91 | -0.08 |
| No. of lateral roots | 6.75 | 0.10 | 0.12 | -1.71 | 0.73 | -0.07 |
| Average lateral root length | 0.61 | < 0.001*** | 0.55 | -0.19 | 0.53 | -0.04 |
| Total root length | 15.96 | < 0.01** | 0.46 | -4.66 | 0.55 | -0.05 |
| Fresh shoot biomass | 0.02 | 0.27 | 0.02 | -0.01 | 0.51 | -0.04 |
| Fresh root biomass | 0.02 | 0.03* | 0.25 | 0 | 0.92 | -0.08 |
| Leaf chlorophyll content | -7.33 | 0.50 | -0.04 | -13.38 | 0.40 | -0.02 |
| Leaf carotenoid content | -2.93 | 0.18 | 0.07 | -1.57 | 0.59 | -0.06 |

**Table S2** Results of linear regression models testing the relationship between concentration of bacterial inoculation and shoot and root dry mass, specific root length, and total root length of *J. vulgaris* seedlings through root inoculation when plants were grown in soil. Presented are estimates, *P* values and R^2^ from linear regression models.

|  | *S. plymuthica* inoculum | | | *P. brassicacearum* inoculum | | |
| --- | --- | --- | --- | --- | --- | --- |
|  | Estimate | P value | R^2^ | Estimate | P value | R^2^ |
| Shoot dry mass | -0.51 | 0.30 | 0.01 | -0.53 | 0.47 | -0.03 |
| Root dry mass | 0.03 | 0.88 | -0.05 | 0.83 | 0.11 | 0.09 |
| Specific root length | -14.80 | 0.14 | 0.07 | -15.34 | 0.21 | 0.04 |
| Total root length | -6.77 | 0.11 | 0.09 | -0.79 | 0.92 | -0.06 |

**Table S3** Results of one-way ANOVA testing effects of inoculum (bacterial cells or supernatant of one of two bacterial strains or control) on primary root length, number of lateral roots, average lateral root length and total root length of *J. vulgaris* *in vitro*. Presented are degrees of freedom (df) and F-values. *** indicates significant difference at *P* < 0.001.

| Root traits | Inoculum | |
| --- | --- | --- |
|  | df | F-value |
| Primary root length | 4, 20 | 12.93*** |
| Number of lateral roots | 4, 20 | 1.78 |
| Average lateral root length | 4, 20 | 13.02*** |
| Total root length | 2, 36 | 10.00*** |

**Table S4** Results of two-way ANOVA testing inoculum (3 levels: two bacterial inoculum and control) and the presence/absence of ethylene inhibitor (AVG) and their interaction on primary root length, number of lateral roots, average lateral root length and total root length of *J. vulgaris* *in vitro*. Presented are degrees of freedom (df) and F-values. *, *** indicates significant difference at *P* < 0.05 or *P* < 0.001.

|  | Inoculum | | AVG | | Inoculum × AVG | |
| --- | --- | --- | --- | --- | --- | --- |
|  | df | F-value | df | F-value | df | F-value |
| Primary root length | 2, 21 | 19.39*** | 1, 21 | 4.83* | 2, 21 | 3.13 |
| Number of lateral roots | 2, 21 | 3.70* | 1, 21 | 0.69 | 2, 21 | 1.55 |
| Average lateral root length | 2, 21 | 49.76*** | 1, 21 | 5.92* | 2, 21 | 3.06 |
| Total root length | 2, 21 | 25.37*** | 1, 21 | 8.59** | 2, 21 | 2.34 |

**Table S5** Results of two-way ANOVA testing in the presence and absence of volatile emitted by one of two bacterial strains (3 levels) and three different amounts of inoculum and their interaction on shoot and root dry mass, and total root length of *J. vulgaris* seedlings per plate *in vitro* (four seedlings together in a Petri dish). Presented are degrees of freedom (df) and F-values. *, *** indicates significant difference at *P* < 0.05 or *P* < 0.001.

|  | Inoculum | | Amount of inoculum | | Inoculum ×Amount of inoculum | |
| --- | --- | --- | --- | --- | --- | --- |
|  | df | F-value | df | F-value | df | F-value |
| Shoot dry mass | 2, 39 | 21.65*** | 2, 39 | 0.04 | 4, 39 | 2.68* |
| Root dry mass | 2, 39 | 13.79*** | 2, 39 | 0.93 | 4, 39 | 3.43* |
| Total root length | 2, 40 | 34.72*** | 2, 40 | 0.38 | 4, 40 | 3.83** |

**Table S6**. Volatile compounds of *S. plymuthica* and *P. brassicacearum* identified from GC-MS analysis.

| Compounds | *S. plymuthica* | *P. brassicacearum* | Molecular formula | Molecular weight (g/mol) |
| --- | --- | --- | --- | --- |
|  | RT | RT |  |  |
| Dimethyl disulfide (DMDS) | 4.019 | / | C_2_H_6_S_2_ | 94.20 |
| Dimethyl trisulfide (DMTS) | 10.909 | / | C_2_H_6_S_3_ | 126.3 |
| Cyclopropane-octyl | / | 13.38 | C_11_H_22_ | 154.29 |
| Methyl 2-hydroxy-4-methyl-4-nitroso-pentanoate (TMS derivative) | / | 15.58 | C_10_H_21_NO_4_Si | 247.36 |
| Acetic acid, 2-(dimethylamino) ethyl ester | / | 16.99 | C_6_H_13_NO_2_ | 131.17 |

**Table S7** Results of two-way ANOVA testing the inoculum (three levels: two bacterial strains and the control), identity of plant species (10 species) and their interaction on primary root length, total root length, and fresh shoot and root biomass *in vitro*. Presented are degrees of freedom (df), F-values and *P* values. *** indicates significant difference at *P* < 0.001.

|  | inoculum | | Plant species | | inoculum × Plant species | |
| --- | --- | --- | --- | --- | --- | --- |
|  | df | F-value | df | F-value | df | F-value |
| Primary root length | 2, 120 | 2.88 | 9, 120 | 5.56*** | 18, 120 | 3.82*** |
| Total root length | 2, 120 | 2.09 | 9, 120 | 9.35*** | 18, 120 | 3.12*** |
| Fresh shoot biomass | 2, 110 | 2.38 | 9, 110 | 11.03*** | 18, 110 | 1.03 |
| Fresh root biomass | 2, 110 | 1.97 | 9, 110 | 5.57*** | 18, 110 | 1.42 |

**Table S8** Genome properties of *S. plymuthica* and *P. brassicacearum*.

| Attribute | *S. plymuthica* | *P. brassicacearum* |
| --- | --- | --- |
| Genome size (bp) | 5,651,564 | 6,996,581 |
| rRNA genes | 6 | 4 |
| tRNA genes | 86 | 68 |
| tmRNA genes | 1 | 1 |
| Protein-coding genes | 5,164 | 5,950 |

**Figure S1.** Mean (± SE) primary root length (A), number of lateral roots (B), average lateral root length (C), total root length (D), fresh shoot (E) and root biomass (F), leaf chlorophyll (G) and carotenoid content (H) in the presence and absence of one of the two bacterial inoculum at varying concentrations *in vitro*. In (A, C, D, F), letters indicate significant differences between the inoculation treatments (*P* < 0.05) based on a Tukey post-hoc test and bars with identical letters are not significantly different. In (B, E, G, H) there was no significant differences (*P* > 0.05) between inoculum in a one-way ANOVA. *P* values of one-way ANOVA are also presented.

**
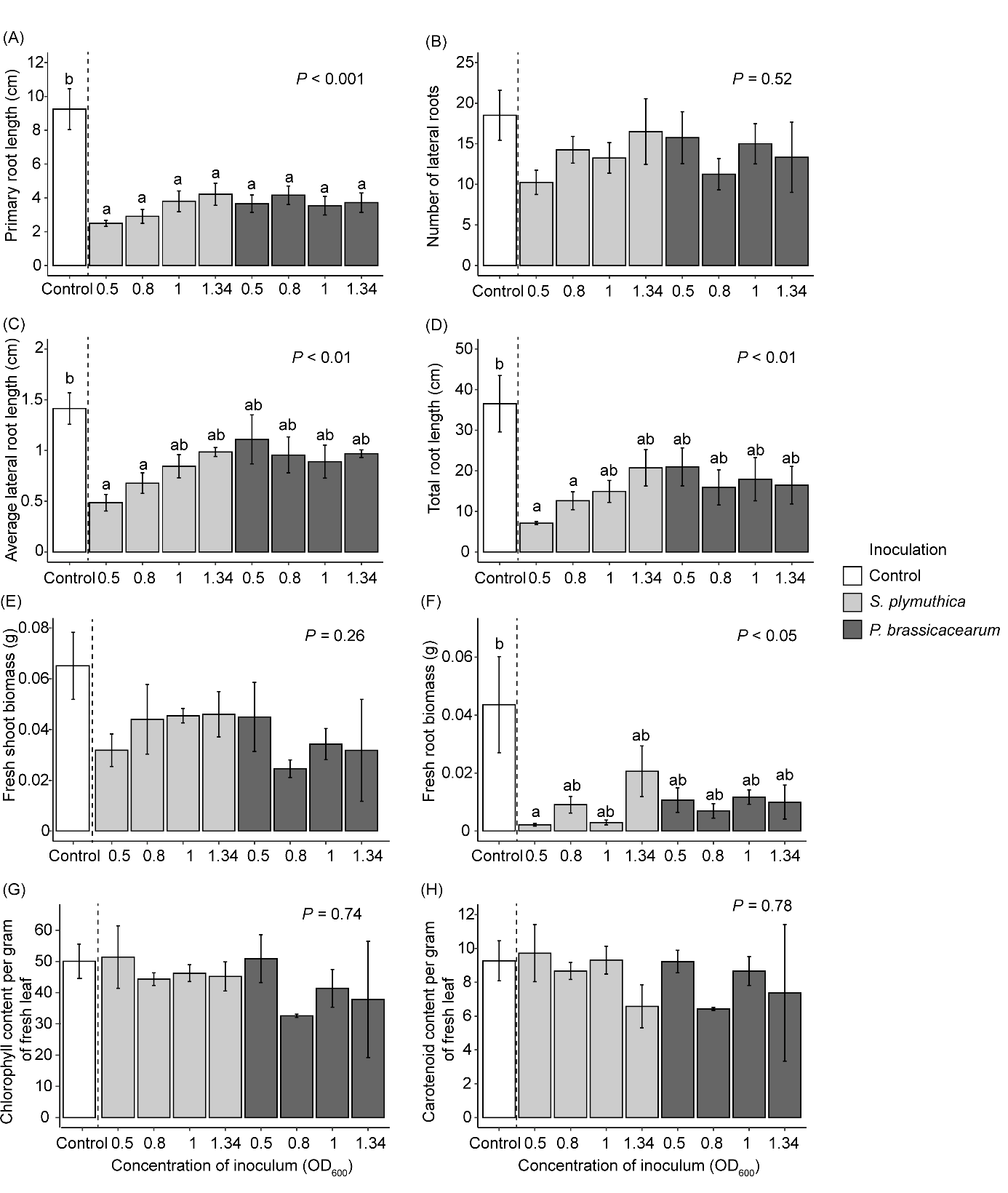
**

**Figure S2.** Preliminary test for hydrogen cyanide (HCN) production. The method was adapted from Ossowicki et al. (2017) and is briefly introduced here: Sterile Whatmann paper (3 mm thick and size 128 × 86 mm) was soaked with a suspension containing 5 mg of both 4,4'-methylenebis (N,N-dimethylaniline) and methyl acetoacetate, copper (II) (Sigma-Aldrich, USA) in chloroform and dried in sterile conditions. One hundred μl of TSB medium was poured into the wells of the 96-well plate. Wells were inoculated with or without 1 μl overnight culture of different bacteria. All bacterial cultures were diluted to OD_600_ = 1. Plate was covered with freshly prepared and dried Whatmann paper and the plastic cover. The plate was incubated 24 h at 25˚C. Change of color from white to blue indicates the production of the hydrogen cyanide (indicated by the red rectangle).


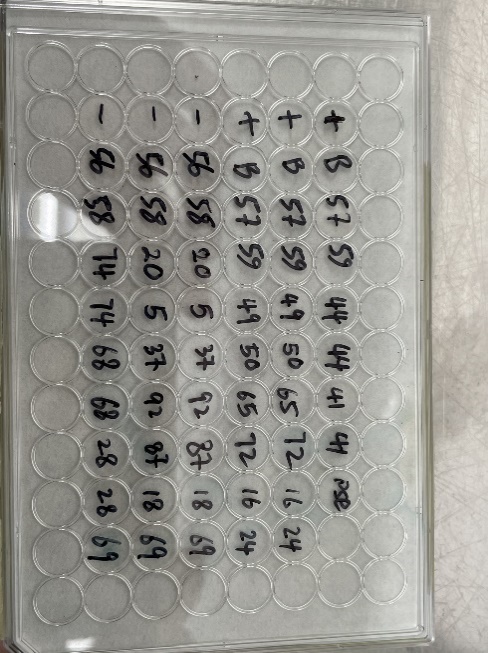


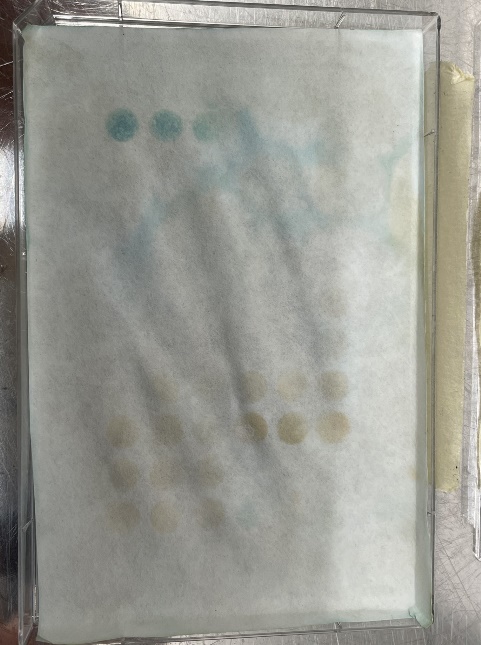

Supplement: fiae116_Supplemental_File [file fiae116_supplemental_file.docx]
